# Unraveling Immunological Dynamics: HPV Infection in Women—Insights from Pregnancy

**DOI:** 10.3390/v15102011

**Published:** 2023-09-27

**Authors:** Carmen Elena Condrat, Dragos Cretoiu, Viorica Elena Radoi, Dana Mihaela Mihele, Mihaela Tovaru, Cristian Ioan Bordea, Silviu Cristian Voinea, Nicolae Suciu

**Affiliations:** 1 Department of Obstetrics and Gynecology, Polizu Clinical Hospital, Carol Davila University of Medicine and Pharmacy, 8 Eroii Sanitari Blvd., 050474 Bucharest, Romania; drcarmencondrat@gmail.com (C.E.C.);; 2 Department of Genetics, Carol Davila University of Medicine and Pharmacy, 8 Eroii Sanitari Blvd., 050474 Bucharest, Romania; dragos@cretoiu.ro (D.C.); viorica.radoi@yahoo.com (V.E.R.); 3 Fetal Medicine Excellence Research Center, Alessandrescu-Rusescu National Institute for Mother and Child Health, 020395 Bucharest, Romania; 4 Department of Dermatology, Carol Davila University of Medicine and Pharmacy, 8 Eroii Sanitari Blvd., 050474 Bucharest, Romania; 5 Dermatology Department, Victor Babes Clinical Hospital of Infectious and Tropical Diseases, 030303 Bucharest, Romania; 6 Department of Surgical Oncology, Prof. Dr. Alexandru Trestioreanu Oncology Institute, Carol Davila University of Medicine and Pharmacy, 252 Fundeni Rd., 022328 Bucharest, Romania; 7 Department of Obstetrics and Gynecology, Polizu Clinical Hospital, Alessandrescu-Rusescu National Institute for Mother and Child Health, 020395 Bucharest, Romania

**Keywords:** HPV infection, pregnancy, immune response, pregnancy outcome

## Abstract

During pregnancy, hormonal and immune adaptations are vital for supporting the genetically distinct fetus during elevated infection risks. The global prevalence of HPV necessitates its consideration during pregnancy. Despite a seemingly mild immune response, historical gestational viral infections underscore its significance. Acknowledging the established HPV infection risks during pregnancy, our review explores the unfolding immunological changes in pregnant women with HPV. Our analysis aims to uncover strategies for safely modulating the immune system, mitigating adverse pregnancy consequences, and enhancing maternal and child health. This comprehensive narrative review delves into the existing knowledge and studies on this topic.

## 1. Introduction

**Figure 1:**
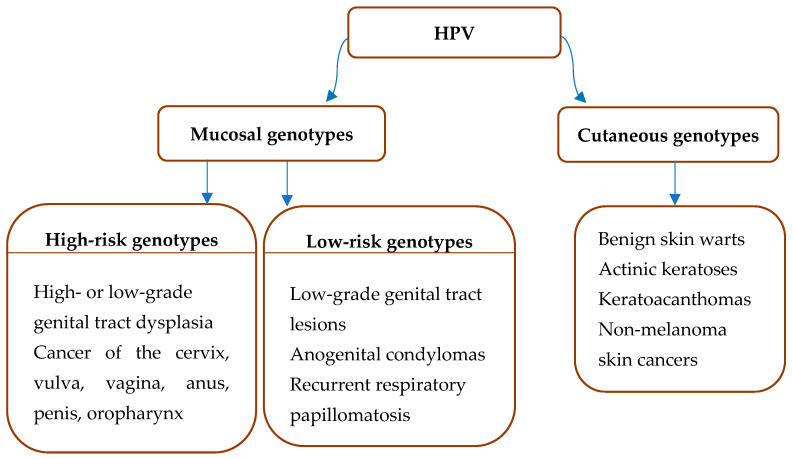
HPV types and infection sites.

Papillomaviruses (PVs) consist of a large group of viruses, of which a subgroup causes the most common sexually transmitted disease, human PV (HPV) infection. HPV has long been reported as the most common sexually transmitted disease worldwide, with studies suggesting that up to 80% of sexually active men and women have contracted the virus at one point in their life [1]. The widespread nature of the infection can be attributed to a variety of factors. Since most cases do not manifest noticeable symptoms, individuals who are infected often remain unaware of their condition and unintentionally propagate the virus [2]. Furthermore, the virus can continue to be transmitted years after the initial infection, as HPV can persist for extended periods without causing symptoms, spanning from several years to decades [3]. However, it is important to highlight that approximately 90% of HPV infections are naturally resolved by the immune response, while only about 10% persist for an extended duration of several years [4]. The lifetime risk of HPV-associated cancer, most commonly cervical cancer, is estimated to be 1–2%. Other affected organs are the vulva, vagina, anus, penis, as well as the oropharynx [5]. Naturally, not all HPV types are capable of causing invasive carcinomas, as the vast majority of HPV genotypes infect the cutaneous epithelium, and only about 40 HPV genotypes affect mucosal membranes (Figure 1) [1]. Cutaneous genotypes commonly result in benign skin warts [6], actinic keratoses [7], keratoacanthomas [8], and non-melanoma skin cancers [9], while they may also be found in healthy skin [10].

Viral infections during pregnancy have long been known to have a major impact on pregnancy outcomes, leading to both fetal and maternal morbidity and mortality. The infections that are traditionally associated with poor pregnancy outcomes, including congenital malformations, spontaneous abortion, premature birth, and low birth weight, are those belonging to the TORCH group (*Toxoplasma gondii*, other agents, rubella virus, cytomegalovirus, and herpes simplex virus). The list of pathogens included in the “other” group has gradually expanded, and it comprises *Treponema pallidum*, hepatitis B virus (HBV), human immunodeficiency virus (HIV), parvovirus B19, and varicella-zoster virus (VZV) [11]. However, more and more agents are being revealed as causes of concern: Zika virus, malaria, and West Nile virus [12]. As the consequences of maternal HPV infection are still ambiguous, it is our aim to provide a synthesis of evidence on the potential mechanisms employed by HPV to circumvent or suppress the immune system.

## 2. HPV Types and Associated Diseases

The Papillomaviridae family is made up of small (52–55 nm in diameter), icosahedral, non-enveloped, or naked viruses that contain a double-stranded circular DNA molecule of a varying number of bases (approximately 8000 base pairs) [13]. The International Committee on the Taxonomy of Viruses recognizes a plethora of different papillomaviruses found in mammals, and classifies them into 53 genera. Of these, only five genera, totaling 225 PVs, can cause infections in humans: *alphapillomavirus*, *betapillomavirus*, *gammapillomavirus, mupillomavirus*, and *nupillomavirus* [9,14]. The highest proportion of known HPV types are included in the genus *gammapillomavirus* (n = 102), followed by *alphapillomavirus* (n = 65) and *betapillomavirus* (n = 54), while the *mupillomavirus* genus only contains 3 types, and the *nupillomavirus* genus only has 1 human PV [14].

Within the *alphapillomavirus* genus, there is a subgroup of 13 HPVs affecting the mucous membranes that the International Agency for Research on Cancer (IARC) has labeled as carcinogenic to humans (IARC Group 1): HPV16, 18, 31, 33, 35, 39, 45, 51, 52, 56, 58, 59, and 66, and are also referred to as high-risk (HR) HPVs [15]. These HPV genotypes are accountable for malignancies of the cervix, vulva, vagina, anus, penis, and oropharynx (Figure 1) [1]. There are 20 different HPV types that are found more frequently in women with cervical cancer than in women with normal cytology [16]. The seven most important HPV types (HPV 16, 18, 31, 33, 45, 52, and 58) are responsible for 90% of all cases of cervical cancer [17,18]. On the other hand, the low-risk (LR) genotypes targeting the mucous membranes, while responsible for condylomata acuminata or benign genital warts, are generally associated with high levels of stigma and shame [19]. Additionally, the *alphapillomavirus* genus also contains several cutaneous HPV genotypes, which can lead to benign skin warts [20]. The HPV genotypes belonging to the genera *beta-*, *gamma-*, *mu-*, and *nupapillomavirus* have an affinity for cutaneous epithelial cells. In addition to cutaneous papillomas and benign skin warts, it has become increasingly evident that *beta*-HPVs play a causative role in the early stages of non-melanoma skin cancers (NMSCs) such as basal cell carcinoma and squamous cell carcinoma, particularly when exposure to ultraviolet radiation is present [21].

Cervical cancer is currently the fourth most common malignancy among women worldwide [22], and recent statistics suggest that about 99% of cases are HPV-related, as genetic predisposition seems to play a meaningless role [23]. Additionally, HPV appears to lie at the root of 90% of anal cancers [24], 50–80% of oropharyngeal cancers [25], 70% of vaginal cancers [26], and 40% of vulvar cancers [27]. Among men, HPV is responsible not only for anogenital and oropharyngeal cancers and warts [28,29,30], but also for decreased sperm motility and issues with sperm morphology, with authors implying that HPV constitutes a key component of male infertility [29,31].

### HPV Structure and Life Cycle 

**Figure 2:**
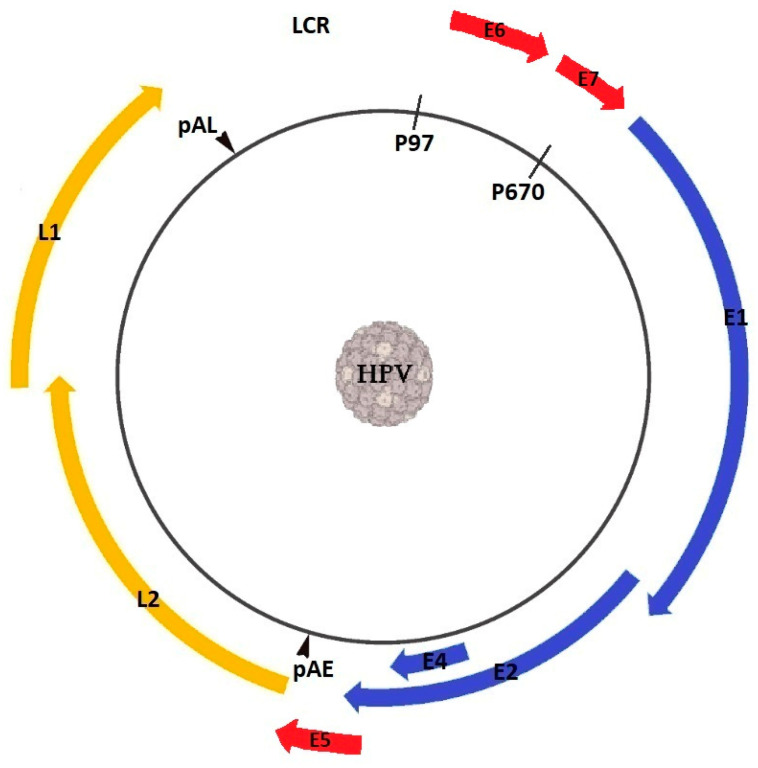
Genome organization of human papillomavirus.

A small DNA-containing virus, HPV replicates and assembles only in the nucleus, infecting the bottom layers of stratified squamous epithelia. The double-stranded DNA molecule is confined in a protein capsid that is made up of two virally encoded structural proteins, namely late 1 (L1), and late 2 (L2) [15]. Depending on genotype, HPV DNA encodes 7 to 10 open reading frames (ORFs) [32,33,34] that can be divided by two polyadenylation (pA) signals, namely early (pAE) and late (pAL), into three functional segments: the regulatory region, referred to as the upstream regulatory region (URR) or long control region (LCR), the early (E) region, and the late (L) region (Figure 2). 

The URR facilitates gene transcription due to it containing transcription factors such as nuclear factor one (NFI), octamer transcription factor 1 (OCT1), and specificity protein 1 (SP1), while also promoting DNA replication through the binding sites for the E1 and E2 proteins [35]. The early region encodes the E1 to E8 proteins, and it is essential for viral replication, while the late region encodes the L1 and L2 proteins [36]. Both E1 and E2 recognize the genome sequence where replication is initiated, while E2 also regulates viral gene transcription and genome segregation during mitotic cellular division [37]. E4 and E5 are thought to be involved in the productive phase of the lifecycle of HPV [38], while the roles of E3 and E8, which are only present in a small number of HPVs, are still under debate [39,40]. E6 and E7 are involved in the immortalization and transformation of the affected cells by targeting oncogenic proteins as well as tumor suppressors, such as p53 and pRb [41,42]. The L1 proteins assemble into capsomers that, coupled with the L2 proteins, organize icosahedral capsids surrounding the viral genome [43]. The expression of the L1 major capsid protein, either alone or in combination with the expression of the minor capsid protein L2, leads to the production of virus-like particles (VLPs), which lack core genetic material but are highly immunogenic, eliciting both humoral and cell-mediated immune responses [44].

HPVs are epitheliotropic viruses that only affect the stratified epithelia of the cutaneous surface, anogenital area, and oropharyngeal mucosa [45]. The few infections that do become persistent are more likely to be the result of a constellation of external and internal host factors, including cigarette and alcohol consumption, risky sexual behavior (early sexual activity, unprotected sex, having multiple sexual partners, or sex trading), medication, genetic traits, and an altered immune response (Table 1) [46,47,48]. 

The life cycle of papillomaviruses is initiated within the basal layers [61]. Still, while HPV initially enters the epithelial basal cells, viral production takes place in the suprabasal epithelial cell layers. In vitro studies show that HPV binds to commonly expressed cell surface or extracellular matrix (ECM) receptors such as heparan sulfate proteoglycan (HSPG) or laminin-5; however, it should be emphasized that, in vivo, the primary site for HPV binding is the basement membrane [62]. Either way, once bound, HPV suffers conformational modifications, affecting both the L1 and L2 proteins, thus enabling the virus to go through the critical stages needed for the viral entry process. Specifically, additional heparan sulfate (HS) binding sites are exposed; the virus then makes contact with the uptake receptor by micropinocytosis, and the viral capsid undergoes uncoating [63,64]. Further on, cyclophilins (Cyps) enable a separation between the L1 protein and the L2-DNA complex, and the latter eventually travels through the trans-Golgi network, with the ultimate goal of nuclear access [65]. Interestingly, unlike other viruses, HPV is internalized at a slow pace, with various studies reporting a prolonged cell surface residence that can span over several hours [66,67,68]. 

Primarily due to the combined efforts of E1, E2, and E6, HPV can establish its genome in the cells comprising the bottom layer of the invaded epithelium. E1 binds to a distinct DNA sequence and is thereafter assisted by E2 in organizing into a hexameric complex with helicase activity [69]. Consequently, DNA unwinding takes place, thus delivering a template for progeny viral DNA synthesis [15,70]. Further on, by increasing the activity of telomerase, E6 ensures cell immortalization [71,72]. Under normal circumstances, during cell division, once a cell leaves the basal compartment for the suprabasal layers, it leaves the cell cycle and enters terminal differentiation [73]. Conversely, cervical epithelial cells and keratinocytes infected with HPV are unable to leave the cell cycle and keep sustaining DNA synthesis and cell proliferation as they ascend, mainly as a result of E7 expression [74]. DNA amplification is then ensured by E4, while L1 prepares to form the icosahedral surface of the virion [32,75,76]. The subsequent DNA encapsidation is ensured by the capsid protein L2 [77,78]. L2 is also required for the infectious process, as it lies behind the viral escape from endosomes and then transports the viral genome to the nucleus [65,79]. 

## 3. HPV Infection during Pregnancy

While the assertion that cancer-causing HPV is almost exclusively sexually transmitted [80] is well-established, recent research suggests that a broader perspective might be warranted. Epidemiological data have shown inconsistencies with the notion that multiple sexual partners, early sexual debut, or oral intercourse are universal prerequisites for HPV transmission and subsequent cervical cancer development [2,81,82,83,84]. It is worth noting that HPV infection can occur after a single sexual partner, and while early sexual debut may elevate the risk of early-onset cervical cancer, it does not preclude the potential for cervical cancer development at later stages.

Additionally, the concept of other modes of transmission, such as horizontal transmission within families or self-inoculation, and their role in HPV infection, deserves careful consideration [85]. A vertical transmission from mother to fetus has time and again been reported. Various studies reveal rates of transmission of up to 80% in cases of HPV-positive mothers [86,87]. Other researchers went further and demonstrated that HPV 16 persists at 6 months of age in up to 60% of infants [88]. However, clinical disease is seldom seen at young ages, with infants rarely developing juvenile-onset recurrent respiratory papillomatosis (JORRP) as a result of HPV 6/11 [89,90]. 

Vertical transmission most probably occurs in the perinatal period, especially at the time of passage through an infected birth canal [91,92,93]. Ascending infection is also possible, particularly in the case of a premature rupture of membranes, but also from infected spermatozoa to the fertilized oocyte following sexual intercourse [94]. HPV DNA has been discovered in the amniotic fluid, with positivity rates fluctuating between 15% and 65% [95,96,97]. Other specimens harboring HPV DNA are neonatal cord blood [93], the oral secretions of newborns [93], and fetal membranes [92]. Recent studies have added maternal breast milk to the routes of vertical transmission, although with little likelihood, by detecting HPV DNA in breast milk [98,99,100]. Placental trophoblastic cells can also be infected, as HPV can complete its life cycle in trophoblasts [101]. Moreover, Boulenouar et al. have shown that HPV 16 negatively impacts the survival, adhesion, and invasion of trophoblast cells [102]. Building on this premise, other observational studies found a link between HPV infection and spontaneous abortion [103], preterm delivery [104,105,106], and stillbirth [107].

Several studies have indicated that vertical transmission is more likely to occur in the case of vaginal delivery [83,97,108]; however, other studies failed to demonstrate a protective role of cesarean birth against perinatal HPV transmission [109,110]. Nevertheless, it is currently believed that the risks associated with a cesarean section outweigh the potential benefits. Surgery is therefore recommended only in rare instances when condylomata acuminata physically obstruct the birth canal or when the laceration of the anogenital warts may lead to hemorrhage [111]. While other modes of transmission are intriguing, it is important to acknowledge that they might not carry the same weight in the context of cervical cancer development as sexual transmission does.

During pregnancy, a latent HPV infection is prone to reactivation, leading to disease recurrence [112], with various studies reporting an overall higher prevalence of HPV infection among pregnant women [113,114,115,116,117]. On this note, Smith compared 69 pregnant and 54 non-pregnant women, and found that HPV prevalence among pregnant women increased with gestational age. They attributed this finding to pregnancy and pregnancy-associated hormones modifying the immune responses [113]. Other studies highlight the increased risk of developing cervical cancer among women of high parity, [118,119]. A 13-year follow-up study in Denmark showed that childbirth increased the risk of developing cervical intraepithelial neoplasia grade 3 or worse (CIN3+) in women with a persistent HPV infection [120]. They linked it to the short-term intense exposure to sex hormones such as estrogen, as estrogen has previously been reported to boost HPV gene expression, promote cell growth in the transformation zone, and impact the cervical immune response [121,122,123,124]. Similarly, Eibye et al. have found higher mortality rates attributed to cervical cancer during or shortly after pregnancy [125]. Likewise, Nobbenhuis et al. have unequivocally demonstrated that pregnant women had associated decreased clearance rates for high-risk HPVs, and they attributed it primarily to a modified humoral immune response [126]. 

Throughout pregnancy, HPV-related lesions such as condyloma acuminata tend to significantly increase in size and number, oftentimes requiring local treatment after the first trimester [127,128,129]. However, in some cases, large lesions may obstruct the urinary tract and/or the birth canal, thus prompting the recommendation of cesarean delivery [127,128,130]. Apart from that, HPV infection has been shown to negatively impact pregnancy outcomes [114,131] and lead to reproductive function abnormalities [132,133]. Specifically, in their study, Wiik et al. have associated maternal HPV infection with premature delivery, preterm prelabor rupture of membranes (pPROM), prelabor rupture of membranes (PROM), and neonatal mortality [134]. Spontaneous abortion has also been thought to be influenced by the presence of HPV during the first trimester [135,136], while other authors have linked preeclampsia to HPV infection [137,138]. On the other hand, Eibye et al. have found higher mortality rates with cervical cancer during or shortly after pregnancy [125]. All these aspects show that HPV infection is a serious health issue that would benefit from further consideration based on the current understanding.

## 4. The Immune Response to HPV under Normal Conditions and during Pregnancy

HPV is successful in evading immune system detection as there is generally no viraemia due to the fact that the primary infection takes place in the basement membrane [139]. Additionally, by infecting cells that are meant to undergo programmed cell death, HPV does not induce any major inflammatory reaction and subsequent immune system alert; therefore, it can carry on replicating unhindered [140,141]. Another evasion strategy consists of the low expression of the oncogenes in the basal layers, whereas the highly immunogenic viral proteins are only present in the uppermost layers of the stratified epithelia [142]. 

During pregnancy, the maternal immune system undergoes major changes, seeking to reach an equilibrium between accommodating the allogeneic fetus and protecting both the mother and the unborn child from pathogens. Systemic changes occurring during this intricate process are primarily governed by the endocrine system, which is responsible for pregnancy-associated hormones such as human chorionic gonadotropin (hCG), human placental lactogen (hPL), estrogen, and progesterone [143]. Studies have suggested a unique behavior of the pregnant immunological system, with pregnant women responding slightly differently to various microorganisms [144], with significant data pointing towards an increased susceptibility to certain viral infections [145,146,147]. Research has demonstrated an increased prevalence of HPV among pregnant women, with most studies attributing it to the hormonal and immune changes characteristic of this state [138,148,149]. Additionally, while cervical neoplasia during pregnancy is not necessarily common, it remains the most frequent cancer among pregnant women. At the same time, HPV-positive women carry the risk of passing it on to their child [148]. As the maternal uterine tissue bears the greatest responsibility when it comes to nurturing and protecting the developing fetus against invading pathogens, we will mostly refer to the maternal–fetal interface, particularly the decidua.

The maternal decidua coordinates the vast majority of local immune changes occurring during pregnancy, as the activation of decidual innate immune cells provides the appropriate environment that can sustain the kind of tolerance needed for the development of the semi-allogeneic fetus. Specifically, leukocytes, T cells, NK cells, dendritic cells, and macrophages populate the inner epithelial layer of the uterus transformed during pregnancy [150]. Adaptive cells are educated so that they acquire an adequate response to the allogeneic trophoblast while also remaining capable of detecting the altered self in the case of a viral infection [151]. The gradual shift from the pro-inflammatory Th1 to the anti-inflammatory Th2 status that typically occurs during pregnancy plays a crucial role in maintaining the maternal immune tolerance towards the allo-antigens expressed by the fetus [152]. Normal pregnancy is further characterized by an overall surge in innate immune cells [153], complement function [154], as well as an amplification of the innate signaling pathways involved in the antiviral response. As an example, studies have shown that the IFN-α-induced signal transducer and activator of transcription 1 (STAT1) signaling cascade is upregulated in decidual NK (dNK) cells, myeloid dendritic cells, and monocytes [153,155,156]. It is noteworthy to mention that the uterus is abundant in NK cells, which gradually increase in number following ovulation and successful implantation. Higher numbers of NK cells can be observed all throughout the second trimester, after which they progressively reduce to almost undetectable levels at term [157]. 

### 4.1. The Innate Immune Response

**Figure 3:**
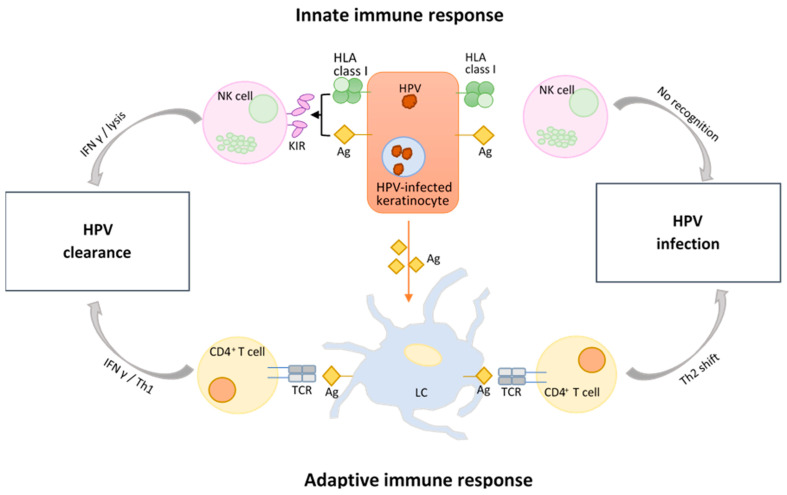
Innate vs. adaptive immune response during HPV infection. HLA = human leukocyte antigen or major histocompatibility complex (MHC); Ag = antigen; KIR = killer cell immunoglobulin-like receptors; NK cell = natural killer cell; IFN γ = interferon γ; Th1/2 = T helper type 1/2 cell; CD4^+^ = cluster of differentiation 4; TCR = T-cell receptor; LC = Langerhans cell. Figure adapted from [164,165].

The innate or non-specific immune response to HPV is conducted by the epithelial cell layer or barrier, the complement system, as well as different phagocytes that engulf antigens and afterwards present them to other immune cells [158]. Antimicrobial peptides (AMPs) also play an important role in the innate immune response as they successfully inhibit viral attachment and replication [159]. However, Langerhans cells (LCs) are the main cells mediating immune surveillance since they are situated in the first line of immunologic defense, both in the skin and the mucosa (Figure 3). As immature dendritic cells (DCs), their main task is to ingest, process, and then present antigens to B and T cells for continued destruction. However, the transformation zone (TZ), where the glandular cells of the endocervix meet the squamous cells of the ectocervix, is associated with lower levels of LCs, and it is the area most likely to develop abnormal cells. Additionally, once squamous intraepithelial lesions (SIL) develop, LC numbers do appear to increase, but their function is altered [160]. The reason for this is because they lack sufficient TNFα expression while simultaneously showing an increase in IL-10 immunosuppressive cytokines, therefore leading to an inadequate activation of T cells [161]. Moreover, while VLPs belonging to HPV16 do elicit the activation of LCs, their being ingested by LCs leads to a dysregulation of the PI3K-Akt signaling pathway, thus contributing to the defective cellular activation [162,163].

AMPs aiding the antiviral response can undergo differential expression during pregnancy. For instance, it has been reported that human β defensin 1 (HBD1) and HBD3 are elevated in the amniotic fluid of women with infection-related preterm labor [166,167]. α-defensins, on the other hand, are known for their anti-HPV properties as they are capable of preventing virion release from cytoplasmic membrane-bounded vesicles as well as decreasing local proinflammatory cytokine production [168]. α-defensin 5 (HD5), found in the genitourinary tract, possesses time-independent anti-HPV activity, as it employs multiple mechanisms against infection. For instance, HD5 prevents the cleavage of the L2 protein by furin, thereby blocking virion escape [169]. Further, it disrupts the detachment of the HPV capsid from the genome and diverts the viral particle towards the lysosome, while also precipitating the destruction of the internalized capsid proteins [170]. Under pregnant conditions, HD5 protects the female reproductive tract against HPV not only within the endocervix, endometrium, and fallopian tubes [171], but also in the chorion and amnion [172,173]. However, during pregnancy, Escribese et al. have recently witnessed a significant drop in the production of α-defensins 1-3, especially in the third trimester, which they linked to the regulatory control of 17-β-estradiol [174]. On a similar note, the antiviral potential of cathelicidins, specifically LL-37, reportedly extends to HPV, with high expressions being identified in both mucosal and cutaneous lesions [175]. During late pregnancy, by inducing the pro-inflammatory NFκB signaling pathway, LL-37 promotes the secretion of tumor necrosis factor-α (TNF-α), IL6, IL8, and monocyte chemoattractant protein 1 (MCP1), thus mediating the proinflammatory response [176]. 

Pathogen recognition receptors (PRRs) are cell surface, cytosolic, and endosomal proteins that detect and initiate an antiviral immune response. They can be observed in a group of cells with non-specific immunity, aiding them in identifying threats via pathogen-associated molecular patterns (PAMPs) and damage-associated molecular patterns (DAMPs) [177]. DAMPs, also referred to as alarmins, are endogenous ligands typically discharged during cell damage and death, and, in large amounts, in cancer [178,179]. PRRs impacted by HPV oncoproteins include toll-like receptors (TLRs), retinoic acid-inducible gene I (RIG-I)-like receptors (RLRs), but also cytosolic detectors such as the cyclic GMP-AMP synthase (cGAS)—stimulator of interferon genes (STING) signaling pathway.

There is some controversy regarding the role of TLR expression in neoplastic cells, as it can be associated with either positive or negative outcomes [180]. More precisely, on the one hand, during HPV infection, TLRs recognize viral DNA and trigger an immune response, and, on the other hand, they can contribute to the transformation of the infected cells by modifying the intracellular signaling cascades [181]. For instance, TLR 4 has previously been shown to be linked with various types of cancer, such as hepatocellular carcinoma [182], gastric cancer [183], prostate cancer [184], and ovarian cancer [185]. Recent studies have shown that HPVs regulate TLR expression and influence TLR signaling pathways, ultimately facilitating persistent infection and carcinogenesis [186]. Following infection, HPV DNA released into the cytoplasmic matrix can be recognized by TLR 4 and 9. TLR 4 identifies the association between HPV and heparan sulfate [187], while TLR 9 detects cytosine-guanine (CpG) fragments within E6 [188]. By initiating the TLR signaling pathways, proinflammatory cytokines and IFNs are then expressed. HPV clearance has been associated with higher levels of TLR 3, 7, 8, and 9 [189,190]. Conversely, TLR 4 has been shown to have an increased expression in more severe HPV-associated cervical lesions, thus attributing it an essential role in the occurrence and progression of cervical cancer [191]. Additionally, it is believed that, by regulating the nuclear factor-κB (NF-κB) and hypoxia-inducible factor (HIF-1α) signaling cascades, TLR 4 boosts the secretion of immunosuppressive cytokines such as transforming growth factor beta 1 (TGF-β1) and IL-6, which contribute to apoptosis resistance [191,192]. On the other hand, following the recognition of HPV DNA by TLRs, especially TLR 9, the expression of interferon (IFN) -α, -β, and -γ is induced, which is a critical antiviral defense system. However, HPV seems to have figured out a way to avoid type I IFN [193]. The transcription of E6 and E7 is only inhibited by IFN-γ, with studies suggesting that E7 actually inhibits signal transduction via IFN-α by binding to interferon regulatory factor (IRF) 9. In doing so, it prevents its translocation to the nucleus, thus impeding the development of the interferon-stimulated gene factor 3 (ISGF-3) transcription complex that binds to the interferon-stimulated regulatory element (ISRE) [194,195]. This is further substantiated by the discovery that E7 mRNA levels are higher in patients that are non-responsive to IFN-α treatment, as opposed to those who respond well to treatment [196]. E7 continues to hinder the IFN signaling pathways by blocking the transactivation function of the tumor suppressor IRF-1, as it is capable of recruiting histone deacetylase at promoter regions [197,198]. 

TLRs are widely expressed in the placenta, with TLR 3, 7, 8, and 9 playing important roles in the antiviral defense. TLR3 recognizes double-stranded viral RNA and activates IRF3, subsequently increasing type I IFN production [199], while TLR 7/8 can distinguish single-stranded RNA viruses. TLR9 prevents viruses from copying and transcribing their genetic code by binding to the unmethylated CpG dinucleotides on their genome [200]. This is especially important during HPV infection, and Hasan et al. have found that the papillomavirus suppresses the function of TLR9 via E7, promoting cervical cancer [201]. In pregnant women, Sánchez-Luquez et al. have found that TLR 7 and TLR 9 together decrease the risk of placental viral infections by observing the impact of TLR 7 and TLR 9 single nucleotide polymorphisms (SNPs) within the genes of these molecules [202]. Similarly, TLR 3 is highly expressed at the trophoblast level during the first trimester. This is an important aspect since, upon the recognition of the double-stranded viral DNA by TLR3, early trophoblasts secrete not only IFN-β but also other antiviral factors, such as anti-microbial factors such as the apolipoprotein B mRNA-editing enzyme-catalytic polypeptide-like 3G (APOBEC3G), Myxovirus resistance A (MxA), secretory leukocyte protease inhibitor (SLPI), and 2′, 5′-oligoadenylate synthetase (OAS) [203,204,205].

Another group of effective viral detectors is embodied by the retinoic acid-inducible gene I (RIG-I)-like receptors (RLRs) which, unlike TLRs, act as intracellular sensors [206]. Upon detecting the RNA virus, RIG-I proceeds to trigger the induction of type I IFN, ultimately increasing the expression of IFN-β [207]. During HPV infection, Chiang and colleagues have recently demonstrated that the RIG-I-mediated expression of ISGs, chemokines, and IFN- β is inhibited by E6. It appears that E6 forms a complex with the ubiquitin ligase tripartite motif containing 25 (TRIM25) and the ubiquitin-specific peptidase 15 (USP15), the latter being an upstream regulator of the former. As E6 promotes the degradation of TRIM25, it ends up preventing the RIG-I ubiquitination, and as a result, HPV manages to evade an innate immune mechanism [208]. 

The stimulator of interferon genes (STING) is another crucial component of innate immunity, as it triggers endoplasmic reticulum stress upon detecting bacterial and/or viral PAMPs [209,210]. Cytosolic viral DNA alerts the cyclic guanosine monophosphate-adenosine monophosphate (cGAMP) synthase (cGAS), which, in turn, activates innate immune signaling [211]. Once activated, cGAS produces a STING agonist, which results in the generation of the autophagosome and the synthesis of chemokines and cytokines with strong antiviral activity [212,213]. The HPV E7 oncoprotein has been demonstrated to be a potent cGAS-STING pathway inhibitor, as it binds to STING by utilizing a Leu-X-Cys-X-Glu (LXCXE) motif and subsequently blocks it [214]. During pregnancy, it seems that STING-dependent autophagy is induced, defending the host cells from viral attacks [215].

Macrophages are important players in both innate and adaptive immunity, as they ingest pathogens and activate lymphocytes along with other immune cells. During HPV infection, it has been reported that the transcription of chemokines promoting macrophage aggregation, such as macrophage inflammatory protein (MIP-3α) and monocyte chemoattractant protein-1 (MCP-1), is inhibited by E6 and E7 [216]. Moreover, Kindt et al. have recently discovered that the macrophage migration inhibitory factor (MIF) is significantly upregulated in HPV-positive cells, due to the synergistic action of E6 and E7. Specifically, they both induce the expression of HIF-1α, thus promoting MIF expression and creating a pro-inflammatory tumor microenvironment [217]. In opposition to their antitumor role, macrophages aggregated in solid tumors inadvertently contribute to tumor progression as they support the proliferation and migration of tumor cells, along with neovascularization [218,219]. A reason for this is that tumor-associated macrophages (TAMs) belong to the M2 immunomodulatory phenotype, exhibiting an increased expression of metalloprotease-9 and vascular endothelial growth factor (VEGF). As HPV-related tumors progress, M2 macrophages become the main population [220,221].

Macrophages increase in number during pregnancy, not only systemically but also at the decidual level, where they make up to a quarter of the local leukocyte population [222]. At the maternal–fetal junction, the responsibilities of macrophages include antigen presentation, trophoblastic debris removal, proangiogenic growth factors secretion, as well as spiral artery remodeling [223]. In their recent work, Ambühl and colleagues have uncovered the fact that HPV is present not only in trophoblast cells but also in the placental macrophages termed Hofbauer cells [224]. Slatter similarly found significant macrophage villous infiltration in HPV-associated lymphohistiocytic villitis, attributing particular adverse pregnancy outcomes to HPV [137].

Other prominent components involved in the immune surveillance are natural killer (NK) cells, a type of cytotoxic lymphocytes with a rapid response to stressed cells. Both E6 and E7 inhibit the production of IFN-γ (Figure 3), as they suppress IL-18 by directly binding to it [225]. In doing so, HPV further prevents the activation of NK cells since IL-18 normally promotes their expansion and enhances their cytotoxicity and antitumor activity [225,226]. Another way in which E6 and E7 impair the function of IL-18 is by degrading the interferon γ-inducible protein 16 (IFI16) inflammasome, thus impeding the inflammatory cell death known as pyroptosis [227]. Cervical tumor cells further weaken the response of NK cells by suppressing the production of cytokines essential for their activation and proliferation. In addition to that, they also release TGF-β and IL-10, both of which have an inhibitory effect on NK cells [228].

Decidual NK cells are unique due to their surface proteins, which abolish their cytotoxic capacity against the allogeneic trophoblast. While it was initially thought that the reason behind this was a lack of cytotoxic granular proteins [229], later studies have shown that dNKs cannot polarize their granular proteins towards the target, in this case —the non-self, trophoblast cells [230]. Moreover, Siewiera and colleagues later demonstrated that dNKs are highly active against virally infected cells [231], but their cytotoxic effect vanishes when targeting infected extravillous trophoblast cells [232]. As Gomez et al. have then found that HPV can be identified in the extravillous trophoblast from spontaneous preterm delivery placentas, it has been hypothesized that the reduced activity of dNK cells can favor persistent HPV infection and, to some extent, placental dysfunction [105,233]. 

### 4.2. The Adaptive Immune Response

The adaptive or specific immune response against HPV enlists the help of B and T cells. B lymphocytes are in charge of the humoral response, which occurs after B cells are stimulated by antigen-presenting cells (APCs) and aided by T helper (Th) cells in maturing and producing specific antibodies. In HPV infection, antibodies are mostly directed towards the L1 major capsid protein and, less commonly, towards E2, E6, E7, and L2 [234,235]. Neutralizing L1 antibodies inhibit either cell surface binding or basement membrane binding, thus preventing virus internalization. T-cell-mediated immunity is believed to be essential in the evolution of HPV infection, as both mucosal and cutaneous lesions are rich in T-cells during the spontaneous regression of tumors [236]. However, only about 50 to 60% of women naturally exposed to HPV become seropositive, and the extent to which this natural protection is efficient against future exposure has been and is still under debate [237,238,239]. Meanwhile, L1 antibodies following vaccination with VLPs demonstrate higher serum levels that persist in the long run [142].

The major histocompatibility complex (MHC) or human leukocyte antigen (HLA) system contains a set of closely related cell surface molecules, whose main role is to bind and display pathogen fragments for lymphocyte recognition. While MHC class I molecules are present on all molecules, MHC class II molecules can only be found on APCs [240]. Evans and colleagues have recently studied the expression of MHC class I and II molecules in HPV-positive and HPV-negative cervical cancer samples. It appears that MHC class I and II molecules were remarkably higher in HPV-positive tumor samples [241]. The same research team had earlier found that HPV-positive cervical cancers have increased CD4^+^ and CD8^+^ T cell activation along with higher lymphocyte infiltration within the tumor microenvironment [242]. Similar results have previously been reported by Gameiro et al. [243], in contrast with studies performed on cell cultures, which report a reduction of MHC class I expression mediated by E7 [244,245]. 

The specific immune response relies heavily on the support of cytotoxic CD8^+^ T cells (CTLs), as they recognize antigens through molecules of MHC class I and proceed to destroy the virus-infected cells. In HPV infection, E5 stimulates CTL activity [246], but it is also capable of downregulating MHC class I, thus diminishing its recognition by CD8^+^ cells [247]. Further on, HPV has developed additional mechanisms in order to evade the cytotoxic cells; by suppressing the expression of the transporter associated with antigen processing 1 (TAP-1), E7 manages to interfere with the assembly of MHC class I proteins, thus inhibiting antigen presentation [241,248]. On a similar note, it has been observed that HIV-infected patients showing decreased T cell counts tend to associate persistent HPV infection with external genital warts and/or intraepithelial neoplasia and carcinoma [249,250,251,252]. Recently, Morrow et al. have further shown that HPV-induced premalignancy can undergo resolution provided that there is an increase in CD137^+^perforin+CD8^+^ T cells specific for the respective HPV genotype. They managed to achieve a two-fold increase in these CD8^+^ T cells following immunotherapy with the VGX-3100 vaccine, thus acquiring therapeutic vaccine-induced immunity [253].

T helper (Th) CD4^+^ cells are central players of the immune system, as they are the ones dictating the direction of the immune response. More specifically, Th cells are in charge of multiple tasks that stretch from immune cell activation to immune response suppression, with implications for both innate and adaptive immunity [254]. Put concisely, there are two subpopulations of Th cells: Th1 cells, associated with the cellular immune response, macrophage repression, and B cell stimulation, and Th2 cells, which stimulate the humoral immune response while supporting B cell proliferation and antibody production [255]. During HPV infection, a disequilibrium between Th1 and Th2 has been associated with various grades of cervical lesions. In intraepithelial neoplasia and invasive cervical cancer, the Th2-type cytokines are dominant, which, coupled with a weak Th1 response (Figure 3), leads to oppressed cellular immunity and the consequential progression of tumor cells [256,257].

The Finnish Family HPV (FFHPV) study, which took place between 1998 and 2001, was aimed at shedding light on HPV dynamics within families by looking at 329 pregnant women. This study showed that T cell activation markers (HLADR^+^CD3^+^CD4^+^) were lower in mothers with persistent HPV infection. HPV-positive mothers also had lower levels of activated suppressor (CD8^+^) and helper (CD4^+^) T cells [258]. However, contrary to these results, Rodriguez et al. found that persistent genital HPV infection was associated with increased CD4^+^ levels [259].

Regulatory T cells (Tregs) modulate the immune response by suppressing cytokine generation and T cell development, having the main role of preventing autoimmunity. Studies have shown that HPV reduces interferon activity via E6 and E7 and, thus, augments the levels of IL 10 and transforming growth factor β1 (TGF-β1) [260]. As a result, Tregs’ expression is enhanced, which also leads to the production of 2,3-dioxygenase (IDO) and Galectin-1 (Gal-1). While IDO is directly toxic to CD8^+^ T cells, Gal-1 promotes tumor angiogenesis by attaching to the vascular endothelial growth factor (VEGF)-receptor 2. In addition to that, Gal-1 increases tumor cell adhesions to endothelial cells, conferring metastatic potential to tumor cells [261,262]. Overall, Tregs inhibit the function of CTLs and create an immunosuppressive microenvironment, which makes it difficult not only for the immune system to successfully counteract HPV but also for immune activating therapies to exert their effects [263]. 

In pregnant women, Tregs populate the decidua and are increased in the peripheral blood, beginning during the first trimester, having the main role of maintaining the allogeneic pregnancy [264]. While low counts have unequivocally been linked to negative outcomes, such as missed abortion, miscarriage, and preeclampsia [265], the trans-differentiation of Treg into Th17 T cells in retaliation to viral exposure can lead to a perturbation of the cortex architecture. More specifically, provided that the uterine immune activation occurs during neurogenesis, a number of unpublished results have indicated that Th17 cells lead to a transient secretion of IL 6/17a, which, once having reached the fetal brain, leads to white matter damage [266].

## 5. HPV Vaccines

Vaccination against HPV has been widely implemented across numerous countries, with multiple studies consistently showcasing its effectiveness in preventing infection and cancer [57,267]. The current prophylactic HPV vaccines utilize virus-like particles (VLPs) generated through the spontaneous self-assembly of 72 pentameric L1 capsomers [268]. Unlike traditional inactivated vaccines, the HPV vaccines approved by the Food and Drug Administration (FDA) belong to a different category. These include the bivalent HPV vaccine (Cervarix, targeting HPV types 16 and 18), the quadrivalent HPV vaccine (Gardasil, targeting HPV types 6, 11, 16, and 18), and the nonavalent HPV vaccine (Gardasil 9, targeting HPV types 6, 11, 16, 18, 31, 33, 45, 52, and 58). The VLPs trigger the production of highly effective neutralizing antibodies, providing robust protection against infection and dysplastic lesions [23]. The current recommendations issued by the Centers for Disease Control and Prevention (CDC) suggest that both males and females from age 9 to 45 should complete a two- or three-dose vaccine series [269]. However, recent studies have shown that even a one-dose regimen is just as efficient in young girls and women (aged 10 to 25), despite eliciting lower antibody responses [270,271,272,273].

During pregnancy, however, the CDC does not recommend vaccination, as the currently available data are insufficient. Since it is not a live vaccine, there should be no increased risks, and in support of this assumption are the manufacturer’s pregnancy registries and phase 3 clinical trials, where the HPV vaccine was inadvertently administered during pregnancy: clinical trial registration on ClinicalTrials.gov, NCT00092521, NCT00092534, NCT00092495, NCT00092547, and NCT00090220 [274,275].

HPV vaccination remains the most effective tool against infection, and, by ensuring primary prevention, it impedes over 90% of HPV-related cancers in both genders [276,277,278]. Additionally, HPV-positive males seem to gain the added benefit of improved semen quality parameters. To this extent, Foresta et al. have discovered that HPV vaccination of infertile men with HPV infection found in semen samples ameliorated sperm motility and anti-sperm antibodies (ASA) titers [279]. Nevertheless, prophylactic vaccination against HR-HPV is not always possible, thus prompting the need for early detection and quick intervention for effective secondary prevention. This is why clinical trials have been looking at carrageenan, a sulfated polysaccharide obtained from red algae, with potent HPV inhibitory effects. Specifically, it has been shown that carrageenan-based lubricants reduce the risk of genital HPV infection in women and also accelerate the clearance of existing HPV infections. While it cannot match the effectiveness of vaccination, the common opinion is that the role of a carrageenan-based gel is to complement HPV vaccination, especially given its ease-of-use, as it can be self-applied [280,281,282,283].

## 6. Conclusions

In summary, HPV stands as the most prevalent sexually transmitted infection, linked to approximately 5% of cancers within the general population and serving as the leading cause of cervical neoplasia among pregnant women. Despite notable strides, HPV vaccination rates have yet to reach the desired levels, with around 60% of the WHO Member States integrating the vaccine into national routine immunization programs [284]. While current vaccines exhibit prophylactic efficacy, their therapeutic potential remains unfulfilled. Thus, an improved grasp on the intricate interactions between HPV and its host holds paramount importance for advancing innovative therapeutic avenues. 

The intricate interplay between innate and adaptive immune responses orchestrates the clearance of HPV infection, albeit to a limited extent, as HPV adroitly employs evasion tactics against immune defenses. Identifying pivotal immune response components and unraveling HPV’s evasive mechanisms are pivotal for optimizing outcomes.

Furthermore, recognizing the potential risks of HPV to fetal development propels the analysis of immune responses during pregnancy as a foundational pursuit. Addressing adverse outcomes such as preterm birth, miscarriage, preeclampsia, intrauterine growth restriction, and premature membrane rupture is conceivable through sustained, widespread vaccination endeavors. Our emphasis on this facet aims to contribute substantially to attenuating the impact of HPV infection during pregnancy, thereby bolstering maternal and child well-being.

In conclusion, this comprehensive review underscores the multifaceted implications of HPV infection, urging heightened vaccination rates, deeper insights into immune interactions, and increased awareness of HPV’s potential repercussions during pregnancy. By collectively addressing these facets, we aspire to usher in a future with improved preventive strategies and overall health outcomes.

## Figures and Tables

**Table 1:** Risk factors associated with persistent HPV infection.

		Source
External Factors		
Lifestyle habits	Cigarette smoking Alcohol consumption Early sexual activity Multiple sexual partners or having a partner with multiple sexual partners Engaging in sex trading Not using a barrier contraceptive method	[49] [50] [51] [52,53,54]
Medication	Long-term use of oral contraceptives Immunosuppressants Not having been vaccinated against HPV	[55] [56] [57]
**Internal Factors**		
Co-infections	HIV and/or other sexually transmitted infections Other HR-HPV genotypes	[58]
Host genetic risk factors	KLF12 gene CTNND2 gene DAP gene	[59]
Defective immune response	Inflammasome genetics Allelic variations of the HLA locus	[48] [60]

KLF12 = Krüppel-like factor 12; CTNND2 = catenin delta 2; DAP = death-associated protein 1; HLA = human leukocyte antigen.

## Data Availability

No new data were created or analyzed in this study. Data sharing is not applicable to this article.
